# Impact of Calorie-Restricted Cafeteria Diet and Treadmill Exercise on Sweet Taste in Diet-Induced Obese Female and Male Rats

**DOI:** 10.3390/nu15010144

**Published:** 2022-12-28

**Authors:** Adam Alvarez-Monell, Alex Subias-Gusils, Roger Mariné-Casadó, Noemi Boqué, Antoni Caimari, Montserrat Solanas, Rosa M. Escorihuela

**Affiliations:** 1 Institut de Neurociències, Universitat Autònoma de Barcelona, 08193 Bellaterra, Spain; 2 Department of Cell Biology, Physiology and Immunology, Faculty of Medicine, Universitat Autònoma de Barcelona, 08913 Bellaterra, Spain; 3 Department de Psiquiatria i Medicina Legal, Facultat de Medicina, Universitat Autònoma de Barcelona, 08193 Bellaterra, Spain; 4 Eurecat, Centre Tecnològic de Catalunya, Technological Unit of Nutrition and Health, 43204 Reus, Spain

**Keywords:** energy restriction, physical activity, metabolic syndrome, sex differences, sucrose, wanting, liking

## Abstract

The goal of the present study was to evaluate the sweet taste function in obese rats fed with a 30% calorie-restricted cafeteria diet (CAFR) and/or subjected to moderate treadmill exercise (12–17 m/min, 35 min, 5 days per week) for 9 weeks. A two-bottle preference test, a taste reactivity test, and a brief-access licking test were carried out when animals were aged 21 weeks; biometric and metabolic parameters were also measured along the interventions. Two separate experiments for females and males were performed. Behaviorally, CAF diet decreased sucrose intake and preference, as well as perceived palatability, in both sexes and decreased hedonic responses in males. Compared to the CAF diet, CAFR exerted a corrective effect on sweet taste variables in females by increasing sucrose intake in the preference test and licking responses, while exercise decreased sucrose intake in both sexes and licking responses in females. As expected, CAF diet increased body weight and Lee index and worsened the metabolic profile in both sexes, whereas CAFR diet ameliorated these effects mainly in females. Exercise had no noticeable effects on these parameters. We conclude that CAF diet might diminish appetitive behavior toward sucrose in both sexes, and that this effect could be partially reverted by CAFR diet in females only, while exercise might exert protective effects against overconsumption of sucrose in both sexes.

## 1. Introduction

Obesity and overweight are two of the biggest health concerns worldwide, with one-third of the population being overweight or obese [1]. The increase in available high-fat and high-sugar food choices has contributed to increased obesity rates [2]. The consumption of these foods and drinks has spiked in recent decades and has been associated with an increase in adiposity, biomarkers of metabolic syndrome (MetS), and associated pathologies [2,3].

Studies have tried to elucidate how an obese phenotype impacts sweet taste. It has been reported in humans that a high BMI is associated with lower perceived sweetness, and with increased liking for sweetness [4]. Preclinical studies have shown that the adipokine leptin has been linked to sweet taste function at several levels, from the taste receptors in the taste buds to the brain reward processing system [5]. It is known that leptin alters sweet taste transduction through the Ob-Rb leptin receptor located in the T1R3 cells of the taste bud that are activated by sweet stimuli [6]. In nonobese humans and rodents, the behavioral responses to a sweet stimulus change with the circadian rhythms of circulating leptin, whilst, in obese individuals, these circadian changes in leptin are absent [7].

Clinically, obesity is treated by improving diet quality and reducing caloric intake, by increasing physical exercise, and by bariatric surgery in severe cases [8]. These interventions have been shown to affect the sweet taste function with contrasting outcomes. For example, weight loss induced through dieting [9] or cognitive therapy [10] has been reported to decrease liking for concentrated sucrose solutions in women, although no differences were observed in other studies [11]. Physical exercise appears to have differential effects on sweet taste depending on whether the training is acute or chronic. Acute bouts of intense aerobic exercise increased the intake of sweet solutions and the reported liking of sucrose in healthy subjects [12,13], suggesting a compensatory effect of the nutrient depletion due to the exercise bout [14], while an increase in self-reported physical activity over 6 months decreased the perceived intensity of sweet solutions [15]. However, it is unclear whether these changes in sweet taste function are a direct result of the exercise itself or of the weight loss associated with it [14]. Nutritional state appears not to be a strong determinant of sweet taste function [16], and studies comparing sweet taste detection thresholds between individuals consuming vegan, vegetarian, or omnivorous diets for decades [17] or low-sugar diets for months [18] did not show altered thresholds. However, it has also been reported that a low-vegetable meal increases sweetness desire 2 h after meal consumption [19], while another study reported no differences in sweet intensity and hedonics after a meal [20].

Several animal models have been developed to study obesity [21]. Among them, the cafeteria diet (CAF)-induced obesity (DIO) model, in which animals are fed several high-fat and high-sugar items of human consumption [22,23], is considered a robust model with high face validity for human obesity [24]. CAF diet induces obesity through hedonic eating and hyperphagia due to the high palatability of its ingredients; additionally, it generates more severe MetS symptoms than other high-fat and high-sugar diets [25,26].

In animal models of DIO, a decrease in motivational behavior toward sweet stimuli, as measured by increased latency to approach the sweet stimuli and/or decreased consumption, has been reported in males [27,28,29]. It has also been reported that diet-induced weight loss in male rats increases preference for low concentrations of sucrose [28]. Furthermore, a decrease in preference for highly concentrated sweet solutions in males has been reported in models of gastric bypass surgery [30,31]. The effects of exercise in animal models and how it affects sweet taste have not been extensively studied. We previously reported a decrease in sugared milk and carbohydrate intake in female Sprague-Dawley rats after 8 weeks of treadmill exercise [32]. Other authors have shown that female mice selectively bred for a high runner phenotype showed decreased consumption of artificial sweeteners when exercised [33]. Sex differences exist in human taste perception, with women being generally more sensitive than men [34,35,36]. This sexual dimorphism is also present in rats, in which females show higher intakes of sucrose solutions than males [37,38].

The responses to sweet stimuli in animals can be interpreted in terms of wanting and liking, which are considered two separate processes [39]. Wanting is considered to be related to how much of a reward is consumed, while liking is related to hedonic impact and to which reward is consumed [40]. Wanting is thought to be a process mediated by the dopaminergic system that encompasses several brain structures processing reward value, such as the mesocorticolimbic dopaminergic system. On the other hand, liking is thought to be mediated by small and localized areas, termed hedonic hotspots, mainly modulated by the opioidergic system and overlapping with areas controlling wanting [39]. These two processes are relatively independent, since it has been shown that mesolimbic dopamine depletion impairs the ability to exert motivational effort while not affecting hedonic reactivity [41], and that elevated dopamine levels increase motivational effort while not affecting hedonic reactivity [42]. However, while wanting can be affected without altering liking, modifications that alter liking almost always alter wanting [40,43].

In this study, we aimed to (i) characterize the effects of CAF DIO upon the sweet taste in terms of wanting and liking, (ii) determine the putative changes in wanting and liking of sucrose solutions due to dietary and exercise interventions based on a calorie-restricted CAF diet (CAFR) and treadmill training in DIO animals, and (iii) analyze the changes in food intake, as well as biometric and metabolic parameters, induced by these interventions. We aimed to determine these parameters in Long-Evans rats, both in females (Experiment 1) and in males (Experiment 2).

## 2. Materials and Methods

### 2.1. Experiment 1

#### 2.1.1. Animals

Sixty female Long-Evans rats (Janvier, France), 23–25 days-old at the time of delivery, were used. Animals were housed in pairs in standard Plexiglas cages, maintained under a 12 h/12 h light/dark cycle (lights on at 8 a.m.) in standard conditions (temperature: 21 ± 1 °C; humidity 50 ± 10%). After 1 week of habituation to the animal room, they were randomly assigned to two groups balancing mean body weight (BW): STD (n = 20, BW = 89.11 ± 1.94 g) and CAF (n = 40, BW = 88.58 ± 1.25 g). The experimental protocol was approved by the Generalitat de Catalunya (DAAM 9978), following the ‘Principles of laboratory animal care’ and was performed in accordance with the European Communities Council Directive (2010/63/EU). 

#### 2.1.2. Experimental Design

**Figure 1:**
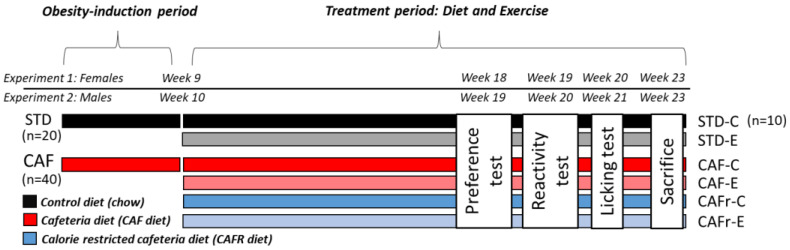
Schematic representation of the experimental layout for experiments 1 and 2 showing groups and the time of behavioral testing.

We used a 3 × 2 factorial design with diet (STD, CAF, and CAFR) and exercise (control C and exercise E) as factors and six experimental groups. After inducing obesity by the administration of the CAF diet for 8 weeks, animals were randomly allocated in pairs to the following groups, balancing mean body weight: STD-C (n = 10, BW = 217.7 ± 4.11 g), STD-E (n = 10, BW = 216.9 ± 4.40 g), CAF-C (n = 10, BW = 264.7 ± 9.67 g), CAF-E (n = 10, BW = 261.2 ± 7.89 g), CAFR-C (n = 10, BW = 259.5 ± 7.51 g), and CAFR-E (n = 10, BW = 263.3 ± 5.98 g). Interventions were maintained throughout the experiment unless otherwise noted. Every week, the animals were weighed, and food intake was determined as the difference between foods provided on Monday and unconsumed the day after. Nasoanal length (NAL) and Lee index $\left(\frac{\sqrt[{3}]{BW}}{LNA}\times 1000\right)$ were recorded every 2 weeks. Behavioral testing started when the animals were 22 weeks old and consisted of the sucrose preference test (week 18), the taste reactivity test (week 19), and the brief-access licking test (weeks 20 and 21). During the 10–12 days after finishing the behavioral testing, the dietary treatment and treadmill sessions occurred as usual. Animals were sacrificed by decapitation at week 23 after 8 h of fasting. Blood was collected, and serum was obtained by centrifugation at 2000× *g* for 15 min at 4 °C and stored at −80 °C until further analysis. The white adipose tissue (WAT) depots (retroperitoneal, RWAT; mesenteric, MWAT; epididymal, EWAT; and inguinal, IWAT) were also removed and weighed. Figure 1 shows a schematic design of experiments 1 and 2.

#### 2.1.3. Diets

During the obesity-induction period, the STD and CAF groups were fed standard chow and CAF diet ad libitum (Supplementary Table S1). From week 9 onward, the CAF diet consisted of (averaged quantities per rat per day) bacon (7 g), biscuits with pâté (5 g), biscuits with cheese (5 g), muffins (7 g), carrots (7 g), jellied sugared milk (40 g), and standard chow (25 g). Food items were administered in a clay pot inside the cage and prepared fresh each day. The average amounts of food and energy administered per rat per day were 96 g and 233 kcal, respectively, and the caloric distribution of the CAF diet was 10.6% protein, 38.3% fat, and 51.1% carbohydrates. We previously used a similar CAF diet in females and showed that it has obesogenic effects [43]. The caloric content of the standard chow (2014 Teklad Global Rodent Diet, Envigo) was 290 kcal per 100 g, and its caloric distribution was 20% protein, 13% fat, and 67% carbohydrates. 

As for the CAFR diet, the amount of each item provided was (averaged quantities per rat per day) bacon (2.4 g), biscuits with pâté (2.5 g), muffins (1.8 g), carrots (6.3 g), jellied sugared milk (4.6 g), and standard chow (7.5 g). The average amounts of food and energy administered per rat per day were 25.1 g and 61.5 kcal, respectively, and the caloric distribution of the CAFR diet was as follows: 11.4% protein, 42% fat, and 46.6% carbohydrates. Food items were also administered in a clay pot inside the cage and renewed daily.

#### 2.1.4. Treadmill Training

Training was performed in a treadmill (Columbus instruments, Columbus, OH, USA) as reported in [44] with some modifications. 

This began on week 9 along with dietary intervention, with daily training sessions from Monday to Friday (5 days/week), and with the intensity raising progressively from 7 m/min in session 1 to 17 m/min in session 9 (Supplementary Table S2). From session 9 onward, all sessions started at an intensity of 7 m/min, increasing progressively until 17 m/min at min 6, which was maintained until minute 30 and then lowered to 8 m/min for the last 5 min. Sessions ended at minute 35, at an approximate total distance run of 591 m. 

#### 2.1.5. Behavioral Testing

- Two-bottle sucrose preference test

To determine the preference for sucrose solutions, a two-bottle preference test was performed. Animals were housed individually the day before starting the test to allow for individualized testing. The exercise intervention was maintained throughout the behavioral test, but all animals were fed only chow to control for interferences between experimental diets and the test results. The test consisted of placing two bottles, one containing a sucrose solution and the other one containing tap water. On the first day, the animals were presented with tap water in the two bottles (250 mL each) placed at opposite sides of the food container cover of the animals’ cage to habituate them to the procedure and to measure water intake for 24 h. The day after and over seven consecutive days, seven concentrations of sucrose solutions were presented in ascending order and tested for 24 h (0.01 M, 0.03 M, 0.06 M, 0.1 M, 0.3 M, 0.6 M, and 1 M). Sucrose solutions were prepared daily using tap water. Water and sucrose intakes were recorded daily, and bottles’ positions were randomly switched between concentrations to avoid lateral preference. *Sucrose preference* ratio was calculated according to the following formula:

$$Sucrose\;preference=\frac{\mathrm{sucrose}\;\mathrm{solution}\;\mathrm{intake}\;\left(g\right)}{\mathrm{water}\;\mathrm{intake}\;\left(g\right)+\;\mathrm{sucrose}\;\mathrm{solution}\;\mathrm{intake}\;\left(g\right)}\times 100$$

- Taste reactivity test

After the two-bottle preference test, the animals were returned to the home cages in pairs. To evaluate hedonic responses to different sucrose solutions, we performed the taste reactivity test according to Shin et al. 2011 [28]. Over four consecutive days, the orofacial responses elicited by four sucrose solutions (0.03 M, 0.1 M, 0.3 M, and 0.6 M) prepared daily using tap water and administered (one each day) in ascending order were recorded. Animals were placed in a cylindrical (29 cm height; 25 cm diameter) Plexiglass cage, and, after 2.5 min, 1 mL of sucrose solution was deposited with a micropipette on the edge of the circular transparent floor. The orofacial expressions were recorded from below using a video camera (JVC, HD Everio GZ-HD10) for 2.5 min from the time the mouth of the animal had the first contact with the solution. Positive hedonic responses (tongue protrusion TP, rhythmic protrusions of the tongue on the midline, and discrete nonrhythmic lateral protrusions of the tongue [45]) were measured as the number of episodes (n) and total duration (s). Behavior was scored by examination of slow-motion videos by a trained experimenter [45].

- Brief-access licking test

To determine the sensitivity and reward value of sucrose, animals were tested on a lickometer (Med Associates Inc., St. Albans, VT, USA). Both the dietary and the exercise interventions were suspended for the duration of the test. The testing procedure encompassed a 4 day habituation period to the apparatus (days 1–4), a habituation session to the test conditions on day 5, and the final test on day 7. The day before starting the habituation period (day 0) the animals were deprived of water. During the habituation period, the animals were placed in the testing chamber and presented with a spout connected to a plastic bottle containing water positioned behind an open slot in the wall of the cage. On days 1 and 2, animals were placed in the chamber and allowed 30 min of uninterrupted water presentation to train them to lick the spout. On days 3 and 4, they were allowed 20 s to lick the spout, and, once licking started, they had 10 s before a shutter blocked access to the spout and a motorized wheel positioned the next spout. Eight bottles containing water were placed in the motorized wheel, and each bottle was presented eight times for a total of 64 trials each session. On day 5, animals were tested with one bottle containing water and the other seven bottles containing sucrose solutions (0.01 M, 0.03 M, 0.06 M, 0.1 M, 0.3 M, 0.6 M, and 1 M). The presentation order was randomized in eight blocks without repetition. When the session ended, the animals were returned to their home cage and allowed access to water. No session was administered on day 6, and the animals were allowed to rest in their home cage. The final test was carried out on day 7 under no water restriction and using the same procedure as day 5. Animals were weighed daily during the test to ensure no animals lost more than 15% of their baseline body weight due to the water restriction. From this test, the number of trials that initiated licking responses, the total number of licks performed in a session, the number of licks performed at each trial, the mean duration of a licking bout (defined as consecutive licks separated by ILIs between 50 and 250 ms), and the interlick interval (ILI) between consecutive licks with a time resolution of 10 ms, which was used to determine the mean ILI distribution for each experimental group, were recorded.

#### 2.1.6. Serum Analyses

Analysis of serum samples was performed as in [44]. Briefly, enzymatic colorimetric kits were used for the determination of serum total cholesterol, triglycerides, and glucose (QCA, Barcelona, Spain). Serum insulin and leptin levels were measured using a mouse/rat insulin ELISA kit (Mercodia, Barcelona, Spain) and a rat leptin ELISA kit (Millipore, Barcelona, Spain). The homeostasis model assessment-estimated insulin resistance (HOMA-IR) was calculated using the following formula [46]: HOMA-IR = glucose × insulin/22.5.

### 2.2. Experiment 2

#### 2.2.1. Animals, Timeline, and Experimental Design

Experiment 2 followed a similar design to experiment 1. For detail on the animals used and the experimental design, see [44]. Of note, the exercise and CAFR interventions started at week 10, the preference test was performed at week 19, the taste reactivity test was performed at week 20, and the brief-access licking test was performed at weeks 21–22. Figure 1 shows the schematic design of experiment 2.

#### 2.2.2. Dietary and Exercise Interventions

A similar CAF diet, CAFR diet, and treadmill exercise to experiment 1 were used in experiment 2. For details on the amounts of food and energy administered per day and treadmill training, see [44]. Of note, the exercise intensity in experiment 2 was set at 12 m/min instead of 17 m/min. This decrease in intensity is because female rats tolerate higher treadmill intensities and are better runners than males [32,47,48].

#### 2.2.3. Behavioral Testing

- Two-bottle sucrose preference test

The test was performed at week 19 using a similar methodology as experiment 1. The corresponding dietary and exercise interventions were maintained during testing.

- Taste reactivity test

The test was performed at week 20 using the same methodology as experiment 1

- Brief-access licking test

The test was performed at week 21 using the same methodology as experiment 1. The exercise intervention was interrupted during the habituation and testing, and the dietary intervention was maintained.

### 2.3. Statistical Analysis

Statistical analysis was performed using the “Statistical package for Social Sciences” (SPSS, version 22, IBM). Biometric and intake data for the first period of obesity induction were analyzed separately with Student’s t-test. Data for the second period were analyzed separately using two two-way ANOVA analyses to determine the effects of the factors diet and exercise: a first two-way ANOVA comparing CAF and STD groups [diet (STD, CAF) × exercise (C, E)] and a second two-way ANOVA comparing CAFR and CAF groups [diet (CAF, CAFR) × exercise (C, E)]. Data comprising several timepoints (BW progression, sucrose intake in the two-bottle preference test, lick score, mean burst size, and mean ILI distribution in the brief-access licking test) were also analyzed with two two-way repeated-measures ANOVAs with time or sucrose concentration as within factors, and diet and exercise as between factors. Sucrose preference and the taste reactivity test data were analyzed with Kruskal–Wallis followed by the Mann–Whitney U test for pairwise comparisons due to unequal variances assessed with Levene’s test. The number of animals that contacted the sucrose solution in the taste reactivity test was analyzed using the chi-squared test.

Mean lick scores data for each group were curve fitted using the following logistic function [49,50]:

$$F\left(x\right)=\frac{a+b}{\left[1+10^{\left(x-c\right)d}\right]},$$

where a is the asymptotic lick response, b is the lower lick response, x is the log_10_ stimulus concentration, c is the log_10_ concentration at the inflection point, and d is the slope of the curve. 

Mean ILI distribution data were curve-fitted to the Gaussian function according to Lin et al. (2013) [51] using the following function:

$$F\left(x\right)=\mathrm{Amp}\cdot {\;\exp}^{-0.5\cdot (\left(\frac{X-\mathrm{Mean}}{\mathrm{SD}}{)}^{2}\right)},$$

where amplitude (Amp) is the height at the center of the distribution, mean is the X value at the center of the distribution, and SD is the standard deviation of the distribution.

All the results are expressed as the mean ± SEM. The level of statistical significance was set at bilateral 5%.

## 3. Results

### 3.1. CAFR Diet Exerted Corrective Effects on CAF-Induced Alterations on Biometric Data in Females

**Figure 2:**
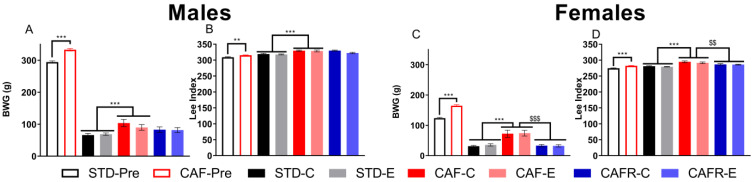
Effects of dietary and exercise interventions on body weight gain (BWG) and Lee index at the end of the obesity induction period (empty bars at the left side of each graphic) and before starting behavioral testing (filled bars). (**A**) BWG in females (week 8, empty bars; week 17, filled bars). (**B**) Lee index in females (week 8, empty bars; week 17, filled bars). (**C**) BWG in males (week 10, empty bars; week 18, filled bars). (**D**) Lee index in males (week 10, empty bars; week 18, filled bars). ** *p* < 0.01, and *** *p* < 0.001 STD versus CAF; $$ *p* < 0.01 and $$$ *p* < 0.001 CAF versus CAFR.

We first analyzed body weight gain (BWG) (Figure 2A,C) and the Lee index (Figure 2B,D), in the first period. We found higher BWG and Lee index in CAF animals than in STD animals in both sexes (*p* < 0.01). Over the second period, this increase in BWG and Lee index in CAF compared to STD was maintained (*p* < 0.001). Moreover, CAFR diet significantly decreased BWG and Lee index compared to CAF in females (second analysis: *p* < 0.01), while, in males, the Lee index tended to decrease (*p* = 0.062) with no significant effect on BWG. No effects of the exercise or the interaction diet * exercise were detected in any parameter in either sex. For details on the progression of BW through the experiment, see Supplementary Figure S1.

### 3.2. CAFR Diet Partially Corrected the Increase in Food and Energy Intake Induced by CAF Diet in Both Sexes

Regarding the dietary intake parameters (Table 1), CAF diet greatly increased the daily total food intake in the first period (*p* < 0.001). This effect was maintained in the second period of the experiments (*p* < 0.001). No effects of exercise or the diet × exercise interaction were detected. In the CAF groups, the energy consumed came mainly from the CAF diet rather than chow (514 kcal versus 86 kcal in males and 518 kcal versus 44 kcal in females). Additionally, throughout the experiments, CAF fed animals decreased chow intake compared to STD fed animals (*p* < 0.001), the diet × exercise interaction in the males’ experiment did not reach significance (*p* = 0.056). 

When comparing CAF and CAFR diets, we found that CAFR lowered the total food intake in both sexes (*p* < 0.001). No effects of exercise or the diet × exercise interaction were observed. When analyzing the chow intake, we found that CAFR increased the energy consumed from chow and decreased the energy consumed from cafeteria diet (*p* < 0.001).

### 3.3. CAFR Partially Corrected the Increase in Serum MetS Biomarkers and Adiposity Induced by CAF Diet in Females

**Figure 3:**
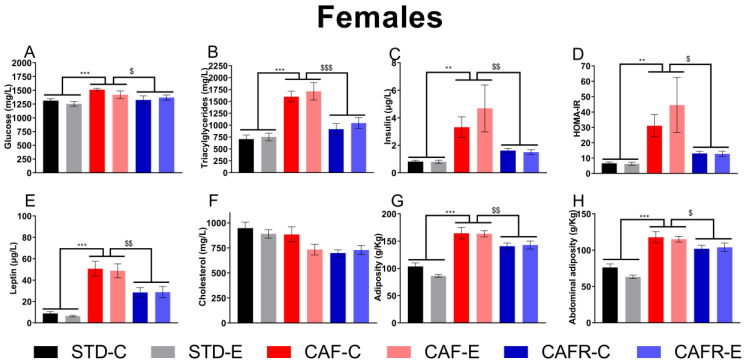
Effects of dietary and exercise interventions on metabolic parameters and adiposity in females. (**A**) Circulating glucose levels. (**B**) Circulating triacylglycerides levels. (**C**) Circulating insulin levels. (**D**) HOMA-IR index. (**E**) Circulating leptin levels. (**F**) Circulating cholesterol levels. (**G**) Total relative (g/kg) adiposity. (**H**) Abdominal (sum of epididymal, retroperitoneal and mesenteric fat depots) relative (g/kg) adiposity. ** *p* < 0.01, and *** *p* < 0.001 versus STD; $ *p* < 0.05, $$ *p* < 0.01, and $$$ *p* < 0.001 versus CAF.

When comparing STD and CAF diets, we found that CAF females increased the circulating levels of glucose (Figure 3A), triacylglycerides (Figure 3B), insulin (Figure 3C), the insulin resistance index HOMA-IR (Figure 3D), and leptin (Figure 3E), but not total cholesterol (Figure 3F) (glucose, triacylglycerides, insulin, and leptin: *p* < 0.001; HOMA-IR: *p* < 0.01; cholesterol: *p* = 0.073). Exercise tended to decrease cholesterol levels (*p* = 0.090). 

On the other hand, CAFR diet decreased glucose, triacylglycerides, insulin, leptin, the HOMA-IR and tended to decrease cholesterol levels compared to CAF (glucose: *p* < 0.05; triacylglycerides: *p* < 0.001; insulin, leptin, and HOMA-IR: *p* < 0.01; cholesterol: *p* = 0.088). No significant diet × exercise interactions and no effects of exercise were detected.

Regarding adipose tissue depots, CAF females showed increased relative total and abdominal adiposity compared to STD females (*p* < 0.001) (Figure 3G,H). CAFR diet decreased total (*p* < 0.01) and abdominal (*p* < 0.05) adiposity compared to CAF diet. No effects of exercise or the diet × exercise interaction were detected in any comparison.

The corresponding data for the males’ experiment were published in a previous article [44]. 

### 3.4. CAF Decreased Sucrose Consumption While CAFR Reverted this Effect in Females, and Exercise Decreased Sucrose Intake in the Sucrose Preference Test

**Figure 4:**
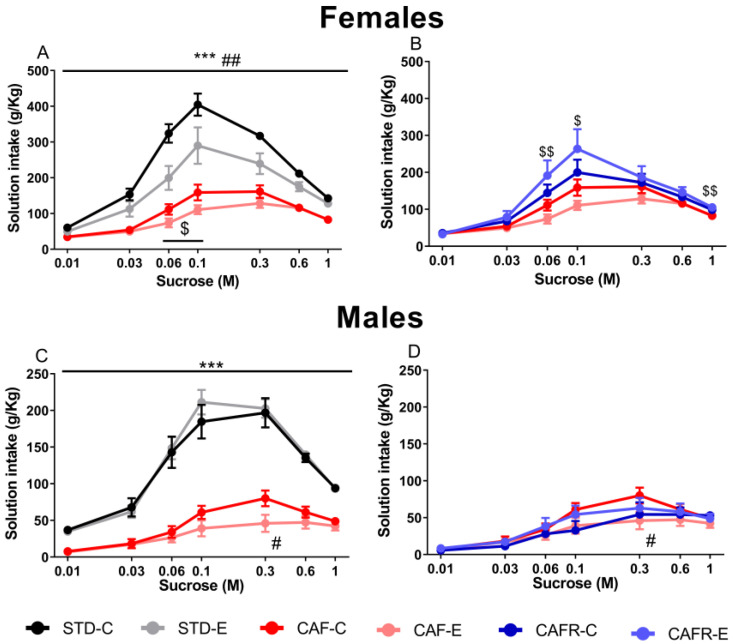
Sucrose intake (g/kg) in the two-bottle sucrose preference test in females and males. (**A**) Sucrose intake for STD and CAF groups in females. (**B**) Sucrose intake for CAF and CAFR groups in females. (**C**) Sucrose intake for STD and CAF groups in males. (**D**) Sucrose intake for CAF and CAFR groups in males. *** *p* < 0.001 STD versus CAF; $ *p* < 0.05 and $$ *p* < 0.01 CAF versus CAFR; # *p* < 0.05 and ## *p* < 0.01 versus non-exercised condition.

Regarding the effects of CAF diet on sucrose preference and intake compared with STD diet, the results showed an inverted-U pattern of sucrose intake in both sexes, increasing between the 0.01 M and the 0.1 M–0.3 M solutions, and then decreasing from that point onward (Figure 4A,C). There was a significant main effect of sucrose concentration (*p* < 0.001), as well as a significant diet × concentration interaction (*p* < 0.001), indicating that the intakes changed with the sucrose concentration in both sexes, and those changes differed between diets. Pairwise comparisons showed that, in both sexes, CAF diet diminished intakes at all sucrose concentrations compared to STD (*p* < 0.001). Exercise in females decreased sucrose concentration intake (*p* < 0.01). Specifically, it decreased the sucrose intake of the 0.06 M solution and tended to decrease the intake of 0.03 M solution [0.06 M, *p* < 0.05; 0.3 M, *p* = 0.079]. The triple interaction of diet × exercise × concentration was not significant in either sex. 

When analyzing the CAFR diet compared to the CAF diet, we found differential sex effects, with increased sucrose intake in CAFR females (*p* < 0.01) (Figure 4B) and no effect on males (Figure 4D). This effect in females was significant for the 0.06 M (*p* < 0.01), 0.1 M (*p* < 0.05), and 1 M (*p* < 0.01) solutions and tended toward significance for the 0.03 M (*p* = 0.052) and 0.6 M (*p* = 0.059) solutions. No effect of the concentration × exercise interaction was observed in either sex. The analysis of the diet × exercise × concentration interaction showed a tendency toward significance in males (*p* = 0.092) but not in females (*p* = 0.108). Decomposition of this interaction revealed that CAF-E males had lower intakes than CAF-C males at the 0.3 M concentration (*p* < 0.05), while CAFR-C and CAFR-E males showed similar intakes.

**Figure 5:**
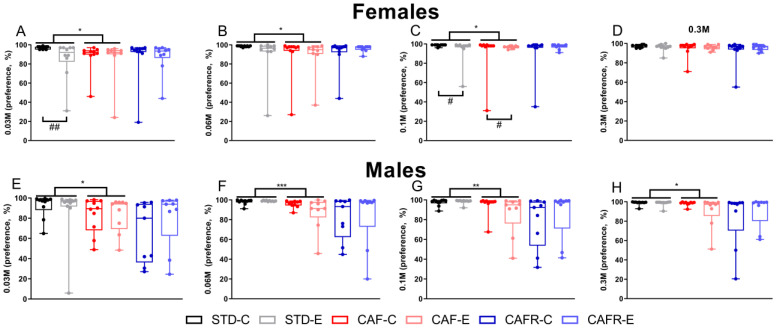
Sucrose preference for different sucrose solution concentrations by group in both sexes. Only solutions in which a significant result was found are shown. (**A**) Preference at 0.03 M in females. (**B**) Preference at 0.06 M in females. (**C**) Preference at 0.1 M in females. (**D**) Preference at 0.3 M in females. (**E**) Preference at 0.03 M in males. (**F**) Preference at 0.06 M in males. (**G**) Preference at 0.1 M in males. (**H**) Preference at 0.3 M in males. * *p* < 0.05, ** *p* < 0.01, and *** *p* < 0.001 STD versus CAF; # *p* < 0.05 and ## *p* < 0.01 versus non-exercised corresponding diet group.

We also analyzed the sucrose preference at each sucrose concentration (Figure 5). Results showed differences in sucrose preference when comparing STD and CAF control and exercised groups. In CAF females, sucrose preference decreased at 0.03 M (*p* < 0.01), 0.06 M (*p* < 0.05), and 0.1 M (*p* < 0.01) compared to STD. In CAF males, sucrose preference also decreased significantly at the 0.06 M (*p* < 0.01) and 0.1 M (*p* < 0.05) concentrations, and tendencies were found at 0.03 M (*p* = 0.072) and 0.3 M (*p* = 0.061) concentrations compared to the STD diet. No significant effect was found between CAF and CAFR in either sex.

Pairwise comparisons showed that CAF females decreased their preference for the 0.03 M (Figure 5A) (*p* < 0.05), 0.06 M (Figure 5B) (*p* < 0.05), and 0.1 M (Figure 5C) (*p* < 0.05) solutions, while CAF males decreased their preference for the 0.03 M (Figure 5E) (*p* < 0.05), 0.06 M (Figure 5F) (*p* < 0.001), 0.1 M (Figure 5G) (*p* < 0.01), and 0.3 M (Figure 5H) (*p* < 0.010) solutions compared to STD. Within diet conditions, exercise decreased sucrose preference in STD and CAF females. Specifically, exercise decreased sucrose preference in STD at the 0.03 M (*p* < 0.01) and 0.1 M (*p* < 0.05) concentrations, and in CAF at 0.1 M (*p* < 0.05).

### 3.5. CAF Decreased Hedonic Reactions to Concentrated Sucrose Solutions in Males but Not in Females

**Figure 6:**
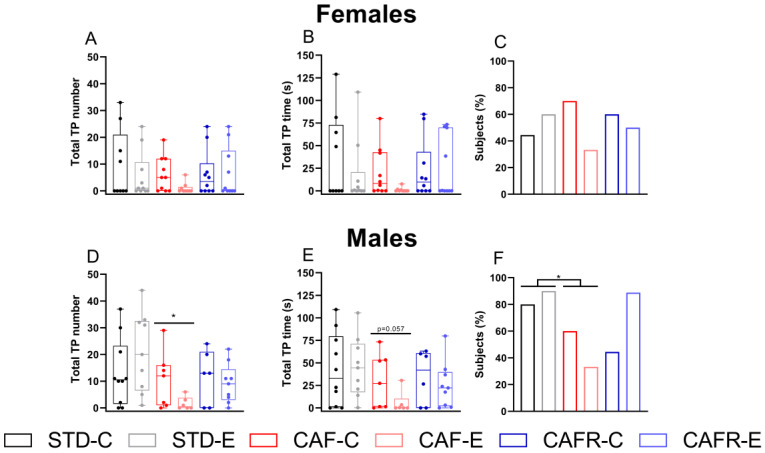
Hedonic responses (tongue protrusions, TP) in the taste reactivity test in both sexes. (**A**) Total number of TPs in females for all sucrose solutions tested. (**B**) Total time spent doing TPs in females. (**C**) Percentage of females that contacted the sucrose solution during the test days and performed at least one TP. (**D**) Total number of TPs in males for all sucrose solutions tested. (**E**) Total time spent doing TPs in males. (**F**) Percentage of males that contacted the sucrose solution during the test days and performed at least one TP. * *p* < 0.05 STD versus CAF.

To determine the hedonic reactions as measured by the tongue protrusions (TP) elicited in response to tasting the different sucrose concentrations (0.03 M, 0.1 M, 0.3 M, and 0.6 M), a taste reactivity test was performed. Results showed that, in males, CAF diet decreased the total number of TPs compared to STD (Figure 6D) (*p* < 0.05). CAF diet also tended to decrease the total time spent performing TPs (Figure 6E) (*p* = 0.094). No significant effects were found in females (Figure 6A–C) when comparing STD and CAF, and no significant differences were found between CAF and CAFR in either sex. The detailed behavior elicited by each sucrose concentration is shown in Supplementary Figure S2.

### 3.6. Licking Test

To determine the reward value and the sensitivity to sucrose concentrations, a brief-access licking test was performed. For details on the training sessions, see Supplementary Figure S3.

#### CAF Decreased the Lick Response in Females and Exercise Decreased It in Both Sexes

**Figure 7:**
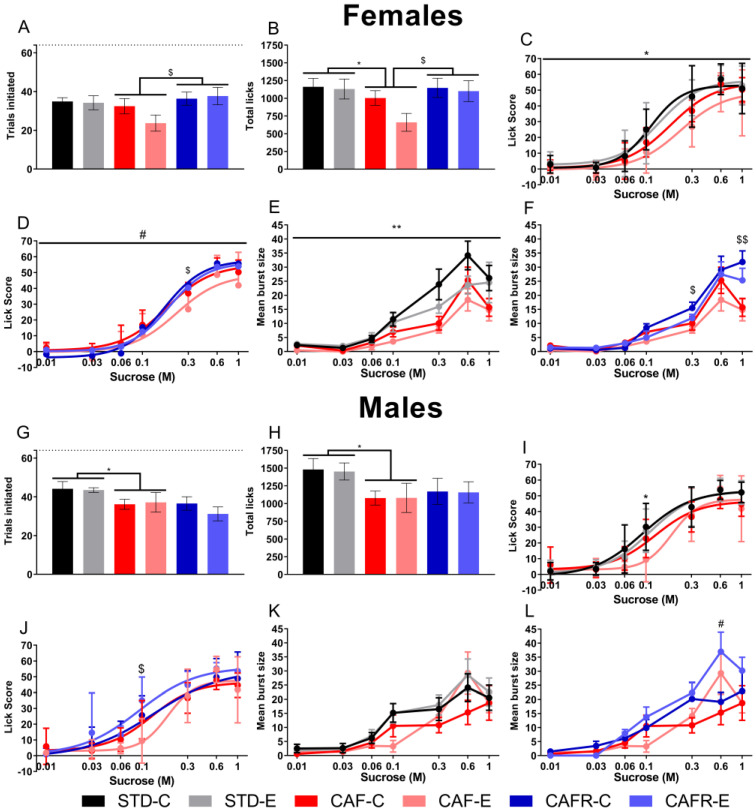
Licking test for both sexes. (**A**) Number of trials initiated in females. (**B**) Total licks performed in females. (**C**) Lick curve in STD and CAF females. (**D**) Lick curve in CAF and CAFR females. (**E**) Mean burst size in STD and CAF females. (**F**) Mean burst size in CAF and CAFR females. (**G**) Number of trials initiated in males. (**H**) Total licks performed in males. (**I**) Lick curve in STD and CAF males. (**J**) Lick curve in CAF and CAFR males. (**K**) Mean burst size in STD and CAF males. (**L**) Mean burst size in CAF and CAFR males. Dashed lines in A and G indicate the total number of trials in a session (64 trials). * *p* < 0.05 and ** *p* < 0.01 STD versus CAF; $ *p* < 0.05 and $$ *p* < 0.01 CAF versus CAFR; # *p* < 0.05 versus non-exercised condition.

The analyses in females showed that CAF diet tended to decrease the number of trials initiated (Figure 7A) (*p* = 0.068) and significantly decreased the total licks (Figure 7B) (*p* < 0.05) compared to the STD diet, with no effect of exercise or the interaction. A main effect of concentration on the number of licks (Figure 7C) was found (*p* < 0.001), but it was unaffected by the interventions. However, the between-subject effects indicated that CAF diet decreased the overall lick score (*p* < 0.05). When analyzing the burst size comparing STD and CAF (Figure 7E), we found a significant main effect of concentration (*p* < 0.001), and no interaction with factors. However, the between-subject effects showed a significance for the factor diet (*p* < 0.01), with exercised animals presenting higher burst size than non-exercised animals.

When comparing CAF and CAFR females, we found an increase in the number of trials initiated (*p* < 0.05) and the total licks (*p* < 0.05) in the CAFR female groups, with no effect of exercise or the interaction. Regarding the lick score (Figure 7D), the analysis revealed that the concentration × diet interaction was significant (*p* < 0.01), indicating an increase of the lick score in CAFR animals at 0.3 M (*p* < 0.05) compared to CAF, with 1 M being close to significance (*p* = 0.055). There were no significant concentration × exercise or concentration × diet × exercise interactions. The test of between-subject effects was significant for the diet × exercise interaction in females (*p* < 0.05), indicating that CAF-E animals performed fewer licks overall than CAF-C (*p* < 0.05), while CAFR-C and CAFR-E animals did not differ. When comparing the burst size (Figure 7F) between CAF and CAFR females, we detected significance for the factor diet (*p* < 0.01), but not for exercise or the interaction, suggesting an increased orosensory stimulation in CAFR females compared to CAF at the 0.3 M (*p* < 0.05) and 1 M (*p* < 0.01) concentrations. Additionally, the between-subject effects revealed a significant effect of diet (*p* < 0.05), indicating overall longer bursts in the CAFR than in the CAF female groups.

The analysis in males of the number of trials initiated and the total number of licks performed in the test session revealed that CAF animals initiated fewer trials (Figure 7G) (*p* < 0.05) and performed fewer licks (Figure 7H) (*p* < 0.05) than STD animals, with no effect of exercise or the diet × exercise interaction on these two variables. When analyzing the lick score at each concentration (Figure 7I), a main effect of concentration was found in males (*p* < 0.001), which was affected by diet and exercise (*p* < 0.05). This translated into a lower response to the 0.1 M solution in CAF-fed animals (*p* < 0.05) and a similar tendency at 1 M (*p* = 0.061) compared to STD. The effect of exercise per se was not noticeable at any concentration. We then analyzed the burst size (Figure 7J), defined as the mean number of consecutive licks with an interlick interval between 50 and 250 ms. Results showed a significant main effect of concentration (*p* < 0.001), indicating progressively longer bursts as concentration increased, but no significant interactions with diet or exercise. This measure is thought to reflect the potency of orosensory stimulation and the perceived palatability of the tastant [52]. 

When comparing CAF and CAFR males, no differences in the number of trials initiated or the total number of licks appeared. We found a tendency toward diet affecting the lick score (Figure 7J) (*p* = 0.066), which translated into an increased lick response by CAFR animals at 0.1 M (*p* < 0.05). No effects on lick response were detected for exercise or the concentration × diet × exercise interaction. The analysis of burst size (Figure 7L) showed an effect of exercise (*p* < 0.010), but no effect of diet or the interaction. Pairwise comparisons between exercise conditions revealed that exercised animals performed longer bursts at the 0.6 M concentration (*p* < 0.05) than non-exercised animals. Additionally, the between-subject factors revealed a significant effect of diet (*p* < 0.05), with CAFR males performing in general longer bursts than CAF males.

Data for the lick score were fitted to a logistic function; the curve fit was good for both experiments with an R^2^ value of 0.763 ± 0.051 for males and 0.819 ± 0.083 for females. 

Data for the ILI distribution by experimental group are reported in Supplementary Figure S4.

## 4. Discussion

In this study, we showed that CAF diet successfully induced obesity both in females and in males, and that CAFR intervention successfully ameliorated this effect. The outcomes of this dietary intervention were greater in females than in males. The CAFR diet in females decreased BW, Lee index, and adiposity and improved metabolic risk factors (decreased serum glucose, insulin, triacylglycerides, and insulin resistance), while CAFR in males decreased only adiposity and leptin levels [44]. CAFR in both sexes decreased the proportion of non-chow foods consumed and increased the proportion of chow consumed, suggesting a healthier food intake pattern. Exercise decreased cholesterol levels in males indicating an amelioration of the MetS risk, but it did not exert noticeable effects upon the biometric variables in either sex.

Regarding the biometric parameters, the obesity induced by CAF diet in this study was similar to that induced in previous studies by our group and others [22,29,53]. Both sexes exhibited similar changes over the obesity induction period, with differences in BW between groups appearing earlier in females than in males. This might be explained by the fact that females presented a higher CAF intake and a lower chow intake than males, which has also been reported in a model of high-fat diet (HFD) [54]. Previous studies reported that rodent males under HFD gained body weight more rapidly than females, which presented a delayed response [54,55]. These results partially contrast with ours; however, the fact that these studies used a HFD while ours used the CAF diet might partially explain the different results we obtained here. Eating patterns differ between DIO diets, with CAF inducing higher intakes than HFD [23,26] and reducing the amount of chow consumed due to the higher preference for palatable foods. Interestingly, females showed a higher preference for cafeteria diet overall than males, in accordance with other studies [54]. 

Another interesting finding of the present study was the differential effect of CAFR diet on decreasing BWG in females but not in males, despite CAFR females maintaining a higher proportion of non-chow food intake than CAFR males. It has been previously reported in mice that, when switching from HFD to chow, females lose body weight faster than males [55], which is in accordance with our results, since our animals were switched from CAF to CAFR diet, which increased chow intake. The CAFR diet shifted the intake of the animals toward a healthier profile that included increased chow intake, which is considered a healthier food choice than CAF, and decreased cafeteria diet intake in both sexes, which is consistent with previous observations in animals fed this diet [29,56]. This might partially mimic the human behavior of eating small portions of palatable energy-dense food while following dietary treatments aimed at increasing the amount of healthy food consumed (here represented by chow). This shift in intake only ameliorated biometric parameters in females, but not in males, despite the adiposity being decreased in both sexes. Metabolically, our CAF intervention resulted in a profile that can be considered characteristic of obesity (hyperglycemia, hypertryacilglyceridemia, hyperinsulinemia and increased insulin resistance, and hyperleptinemia). These consequences of CAF diet have already been widely reported by us and others [25,26,29] and are indicative of the development of MetS in these animals. 

Regarding the metabolic and hormonal parameters, the effects of CAFR diet seemed to differ between sexes, with females showing an attenuation in the levels of circulating glucose, TAGs, leptin, and insulin, as well as the HOMA-IR, while, in males, only the cholesterol levels decreased as a result of CAFR diet [44]. This might be explained by the greater responsiveness that females present to CAFR dieting.

Exercise did not modulate the intake, and no differences were observed between exercised and control animals in food intake, this is also consistent with other reports in which exercise of a similar intensity was unable to modify energy or food intake [47,57]. In line with this, we also detected that exercise had no noticeable effect on either the biometric parameters or the metabolic and hormonal profile in either sex. While similar interventions to the one used here have shown a 25% reduction in body weight gain in adult Sprague-Dawley rats [57], it seems that greater intensities or voluntary exercise rather than moderate treadmill training are more effective at inducing weight loss [58,59]. Another factor that might influence this parameter is the choice of rat strain; in the present study, Long-Evans animals were used, while, in Leigh et al. (2020) [57], Sprague-Dawley animals were used. The Long-Evans rat strain is known to be more active than Sprague-Dawley [60]; this might imply that higher exercise intensities were necessary in Long-Evans than in Sprague-Dawley in order to generate significant changes. Even in the absence of effects on those variables, exercise produced new and noticeable behavioral outcomes, decreasing sucrose consumption in both sexes and decreasing the palatability of sucrose in males.

In this study, we performed three tests which act concurrently to provide information about several aspects of ingestive and motivational behavior: the two-bottle sucrose preference test, the taste reactivity test, and the brief-access licking test. In the preference test, a reduction in sucrose intake can be interpreted as anhedonia and decreased appetitive behavior [61]. Similarly, in the taste reactivity test and the brief-access test, the number of animals initiating the behavior and the number of trials initiated, respectively, can also be interpreted as a measure of appetitive behavior [49]. In the taste reactivity test, the hedonic reactions elicited by the tasting of sucrose are considered a measure of how much the animals liked the stimulus [40,46], and a similar interpretation can be made from the preference index in the preference test [40]. Lastly, in the brief-access test, the lick responses at each sucrose concentration can be interpreted as a measure of the ingestive or consummatory behavior elicited by the tastant [47,48,49]. The length of the licking episodes performed at each sucrose concentration is a measure of the orosensory feedback power and the perceived palatability of that sucrose concentration [52,62].

The behavioral results indicated that “wanting” (i.e., how much of a reward is consumed) was affected by CAF diet. This was observed as a decrease in sucrose intake in the sucrose preference test in both sexes, a decrease in trials initiated in the brief-access test, and fewer CAF-fed animals tasting the stimulus in the taste reactivity test, compared to the STD-fed animals. These three results revealed a decrease in approaching behavior to the rewarding stimulus [49], which is consistent with previous reports in animals fed a CAF diet [27]. This decrease in approaching behavior in the brief-access and taste reactivity tests could result in fewer approaches in the preference test, which could explain the lower sucrose consumption. Altogether, this could be interpreted as diminished appetitive behavior, in part dependent on the dopaminergic system [63]. Concurrent with our results, HFD induced obesity in Long-Evans rats, impaired mesolimbic dopamine function, and decreased the number of operant conditioned responses to sucrose [63]. Moreover, chronic HFD in rats was related to a downregulation of dopamine and opioid receptors in the mesolimbic system [64,65]. Additionally, downregulation of D1 and D2 receptors was observed after chronic CAF diet, and this effect was greater in females than in males [66]. Dopamine release in the nucleus accumbens (NAcc) was shown to be increased after sucrose consumption in sucrose-naïve rats [67]. HFD diet decreased tyrosine hydroxylase-positive neurons in the dopaminergic nigrostriatal pathway [68], and a decrease in dopamine release in the NAcc has been described after intake of milk enriched with 5% fat [69]. The decreased expression of dopamine receptors coupled with the decrease in dopamine synthesis and release in obese animals might imply a devaluation of the reward value of sucrose [70]. 

Another mechanism that might reduce sucrose intake in the preference test is the development of a depressive state that induces anhedonia in obese animals [71,72]. In fact, in a modified version of the sucrose preference test, in which animals are administered 1% sucrose (0.03 M) for 16 h, a diminution in sucrose intake is used to measure anhedonia [61]. In obese rats, the decrease in sucrose intake in a sucrose preference test was interpreted as anhedonia and related to increased adiposity and leptin levels [73]. In humans, obesity and depression are highly comorbid [74], and weight loss due to calorie-restricted diets has been reported to improve depression scores [75]. Several mechanisms have been proposed to link obesity and depression, including a mild level of chronic inflammation characteristic of obesity, chronically increased proinflammatory interleukin (IL-2) levels, and leptin signaling [76,77].

The results regarding appetitive behavior in CAFR-fed animals showed increased sucrose consumption in the preference test and more trials initiated in the brief access licking test compared to CAF diet only in females. This might imply a functional improvement in the dopaminergic system and a partial reversal of wanting compared to CAF diet. In males, there were no effects of CAFR diet on appetitive behavior, with the values of these parameters being similar to those of CAF-fed animals. Therefore, females seemed to be more responsive to the dietary treatments than males regarding appetitive behavior. 

This recovery of appetitive behavior in CAFR-fed females might be related to the amelioration of the obese phenotype. Of note, people in the process of weight loss through calorie-restricted diets showed an amelioration of depression scores [75]; in another study, an improvement of depression scores was positively correlated with a decrease in body weight [78].

With regard to the exercise intervention, a new finding to our knowledge is that treadmill exercise decreased sucrose intake (i.e., appetitive behavior) in the preference test. This result is in line with several studies indicating that exercise might produce functional changes in the dopaminergic mesocorticolimbic pathways that make the animals less susceptible to the rewarding effects of drugs of abuse [79,80]. For example, voluntary exercise on a running wheel decreased self-administration of cocaine in rats [79], and the treadmill exercise in mice decreased MDMA conditioned place preference [81]. In the same way, exercise might have similar effects on sucrose intake, being protective against overconsumption of sweet drinks since the intake of both sucrose and drugs of abuse activates similar pathways in the brain [82]. Exercise has also been reported to increase D2R expression and protein levels in the NAcc [83,84], although conflicting reports have been published [85]. In older humans undergoing regular aerobic exercise at 40–80% of maximal heart rate (walking, jogging, or cycling) for 6 months, D2R availability, measured by PET scanning, was positively correlated with VO_2_ max [86], indicating that physical fitness improves dopaminergic function. Additionally, treadmill exercise at a similar intensity (15 m/min, 40 min) to the one used here induced an increase in dopamine release in the NAcc after voluntary intake of milk enriched with 5% fat in HFD rats [69]. 

Regarding liking (i.e., hedonic impact or which reward is consumed), we detected a decrease in sucrose liking in CAF-fed animals, as evidenced by decreased sucrose preference in the two-bottle preference test and decreased hedonic reactivity in the taste reactivity test. This effect was greater in males than in females since males presented decreased preference in more concentrations than females, and CAF females did not present a significant reduction in positive hedonic reactions. As stated above, liking is thought to be mediated mainly by the opioid system [87] and to be controlled by small regions in the brain [39] termed hedonic hotspots. It has been shown that chronic CAF diet induces a decrease in opioid receptors in the NAcc in males but not in females [66]. In Fam et al. (2022) [27], a similar CAF diet intervention resulted in no changes to liking of sucrose. However, the intervention in Fam et al. (2022) [27] started in adult rats, while ours started post weaning, which might indicate that changes in liking are only present when CAF diet is started early in life. 

Regarding the CAFR diet, results showed no effects of this intervention on liking since both preference and hedonic reactions were not different between CAFR and CAF animals.

Exercise did not affect the hedonic reactivity in the taste reactivity test. The apparent reduction seen in CAF exercised animals compared to CAF controls can be explained by the lower number of CAF exercised animals that initiated behavior, skewing the result toward lower hedonic reactivity. This lack of effectiveness of exercise upon hedonic reactivity is consistent with previous reports of treadmill exercise having a minimal impact on opioidergic signaling [88], considered crucial in the processing of liking [39].

Molecular determinations of the dopaminergic and opioidergic systems are beyond the scope of this work; however, future research should emphasize this aspect, especially in animals fed the CAFR diet.

Consummatory behavior can be evaluated in the brief-access test through the lick score (difference between licks at each concentration and licks to water). Results showed that the CAF diet in males decreased the lick score at intermediate concentrations (0.1 M), with no changes at higher concentrations. This might imply that obese animals present lower consummatory behavior at these concentrations than nonobese animals, putting obese animals at risk of overconsuming at higher concentrations. CAFR diet reverted this by increasing the lick score at the 0.1 M compared to CAF-fed animals, aligning their phenotype more closely with the STD-fed condition.

We detected that exercise decreased the lick score; this effect might act in conjunction with the decreased appetitive behavior in exercised animals explained above to provide additional protection against overeating in exercised subjects.

Perceived palatability of sucrose in the brief-access test is evaluated through the burst size. This parameter is considered to indicate the potency of the orosensory positive feedback and to reflect perceived palatability [52,62]. We found a decrease in burst size in CAF-fed animals and more specifically in the CAF females, indicating a decreased perceived palatability in obese animals. One possible mechanism that might explain this decrease in palatability is a dysregulation of leptin signaling. The leptin receptor is present in the neural pathway relaying taste information from the tongue to the SNC. It has been shown that leptin administration induces hyperpolarization of taste receptor cells [89] and suppresses the response of the peripheral taste nerves to sucrose [90]. Our results showed that serum leptin levels increased in CAF groups, to a greater degree in females than in males (653% increase in females and 224% increase in males compared to the corresponding STD group). This difference in leptin levels between sexes suggests that the suppression of the signal transduction for sweet taste could be greater in females than in males. This would be consistent with the greater decrease in perceived palatability we detected in the brief-access licking test in females than in males. 

Regarding the effects of the CAFR diet, our results show that this intervention corrected the effects of CAF diet on the perceived palatability in females, with the burst size increased in CAFR females compared to CAF females, but not in males. This result is also consistent with the different serum leptin levels in both sexes since the decrease in them was greater in CAFR-fed females than in males (57% versus 83% reduction compared to the corresponding CAF group), suggesting an attenuated suppression of the signal transduction for sweet taste in females [89,90].

Studies in humans have reported that, in women, weight loss induced through either low-fat or low-carbohydrate diets shifted liking for sweet food toward lower concentrations compared to baseline [9]. In another study, obese women instructed to reduce their total caloric intake and to increase their physical activity decreased BW and BMI and shifted their perceived palatability of sucrose toward lower concentrations [10]. Considering these results in humans, although the CAF intervention was interrupted in the females’ experiment, our animals cannot be considered in a fasted state during the behavioral testing since all animals had access to food. Furthermore, although the CAF and CAFR diets varied in the amount of food provided, they had the same food items and a very similar macronutrient composition, indicating that the diet composition per se should not be a determinant of sweet preference or liking. The results from human studies might suggest that weight loss rather than changes in feeding habits are necessary to revert the effects of the obese phenotype on perceived sweetness.

Furthermore, exercise also affected the perceived palatability of sucrose, increasing it. Although this effect was present in both sexes, it was only significant in males, in which exercised animals presented the highest burst size for the 0.6 M solution, while, in controls, the highest burst size was for the 1 M solution. As stated above, leptin is a suppressor of sweet taste [89,90], and the fact that exercise in CAF males increased serum leptin levels might explain the increased detection threshold. This result indicates that exercise may change palatability and switch it toward lower sucrose concentrations, which would be in line with the reduced sucrose intake observed in the preference test, suggesting a protective mechanism of exercise against overeating.

One of the limitations of this study was the lack of a mechanistic characterization of the processes mediating the reported effects. Future studies should aim at characterizing the effects of CAFR and the combined CAFR and exercise intervention on the dopaminergic and opioidergic systems, both at rest conditions and as a response to sucrose intake, as well as leptin receptor expression and dysregulation of leptin signaling in the neural pathway relaying sweet taste from the tongue to the SNC.

## 5. Conclusions and Final Remarks

In this study, we showed that the administration of a calorie-restricted cafeteria diet promotes healthy eating habits, which attenuated the adverse effects of ad libitum CAF diet to a greater degree in females than in males. This study provides the first characterization on the effects of CAFR diet on obese females. To our knowledge, the behavioral characterization of sweet taste behavior presented here in male and female rats is one of the most complete to date. Furthermore, this allows us to conclude that exercise might decrease the perceived sweetness, appetitive behavior, and sucrose consumption in both sexes, whereas CAFR in females increased appetitive behavior which might be indicative of an amelioration of the depressive state associated with obesity.

## Figures and Tables

**Table 1:** Intake parameters in males and females during the obesity-induction period (STD-Pre and CAF-Pre columns) and for the six experimental groups during the interventions period of the two experiments.

		STD-Pre	CAF-Pre	STD-C	STD-E	CAF-C	CAF-E	CAFR-C	CAFR-E
Total Food intake (g/Kg)	Males	90.7 ± 2.48	265.3 ± 24.33 ***	42.95 ± 2.87	46.18 ± 2.63	111.93 ± 13.74 ***	109.26 ± 17.15 ***	78.74 ± 7.73 $$$	76.71 ± 6.61 $$$
	Females	88.9 ± 3.05	229.8 ± 30.76 ***	56.23 ± 4.09	56.23 ± 2.38	130.9 ± 13.31 ***	123.3 ± 16.9 ***	62.03 ± 6.69 $$$	46.5 ± 7.11 $$$
Chow energy intake (Kcal/Kg)	Males	263.0 ± 7.2	86.3 ± 14.4 ***	124.5 ± 8.33	133.9 ± 7.61	24.5 ± 8.21 ***	22.8 ± 10.73 ***	47.4 ± 7.77 $$$	47.3 ± 9.53 $$$
	Females	257.9 ± 8.86	44.8 ± 13.54 ***	163.08 ± 11.87	163.07 ± 6.92	15.61 ± 5.20 ***	22.28 ± 9.31 ***	41.29 ± 6.5 $$$	45.04 ± 10.24 $$$
Non-chow energy intake (Kcal/Kg)	Males	-	514.7 ± 9.96	-	-	233.1 ± 6.20	231.1 ± 6.86	129.2 ± 5.07 $$$	126.4 ± 4.02 $$$
	Females	-	518.7 ± 7.97	-	-	283.4 ± 11.89	275.9 ± 11.52	139.1 ± 4.17 $$$	140.6 ± 3.25 $$$
Chow intake (% of total energy intake)	Males	100	14.38 ± 0.47 ***	100	100	9.61 ± 1.07 ***	8.99 ± 1.31 ***	26.94 ± 1.36 $$$	27.22 ± 1.67 $$$
	Females	100	7.96 ± 0.55 ***	100	100	5.32 ± 0.97 ***	7.30 ± 1.26 ***	22.92 ± 1.71 $$$	24.16 ± 2.10 $$$
Non-chow intake (% of total energy intake)	Males	-	85.62 ± 0.47	-	-	90.39 ± 1.07	91.01 ± 1.31	73.06 ± 1.36 $$$	72.78 ± 1.67 $$$
	Females	-	92.04 ± 0.55	-	-	94.68 ± 0.97	92.70 ± 1.26	77.08 ± 1.71 $$$	75.84 ± 2.10 $$$
Simple sugars (Kcal/Kg)	Males	-	167.6 ± 4.25	-	-	72.8 ± 5.18	69.87 ± 6.03	36.91 ± 2.04 $$$	34.75 ± 1.83 $$$
	Females	-	157.3 ± 6.03	-	-	84.95 ± 4.67	81.43 ± 5.57	31.76 ± 2.28 $$$	33.69 ± 2.30 $$$

Averaged daily total food, chow, non-chow cafeteria diet, and simple sugar intake relativized to BW (kg) or as a percentage of total energy intakes from chow and non-chow. *** *p* < 0.001 STD versus CAF; $$$ *p* < 0.001 CAF versus CAFR.

## Data Availability

The datasets generated and/or analyzed during the current study are available from the corresponding authors on reasonable request.

## Supplementary Materials

The following supporting information can be downloaded at https://www.mdpi.com/article/10.3390/nu15010144/s1: Figure S1: Body weight progression from the start of the experiment to the behavioral testing for experiments 1 and 2; Figure S2: Detail of the TP number and time at each concentration tested; Figure S3: Licking variables during training (sessions 1–5) in the brief-access licking test; Figure S4: ILI distribution in the test session of the brief-access licking test. Table S1: CAF diet composition for the first period of experiment 1; Table S2: Detailed treadmill training protocol from session 1 to 9 for experiment 1.
