# Supplementary material for: Impact of Calorie-Restricted Cafeteria Diet and Treadmill Exercise on Sweet Taste in Diet-Induced Obese Female and Male Rats
Source: Nutrients. 2022 Dec 28;15(1):144. doi: 10.3390/nu15010144 (PMC9823820; doi:10.3390/nu15010144)
Supplement: Supplementary file 1 [file nutrients-15-00144-s001.zip › nutrients-2106676-supplementary.pdf]

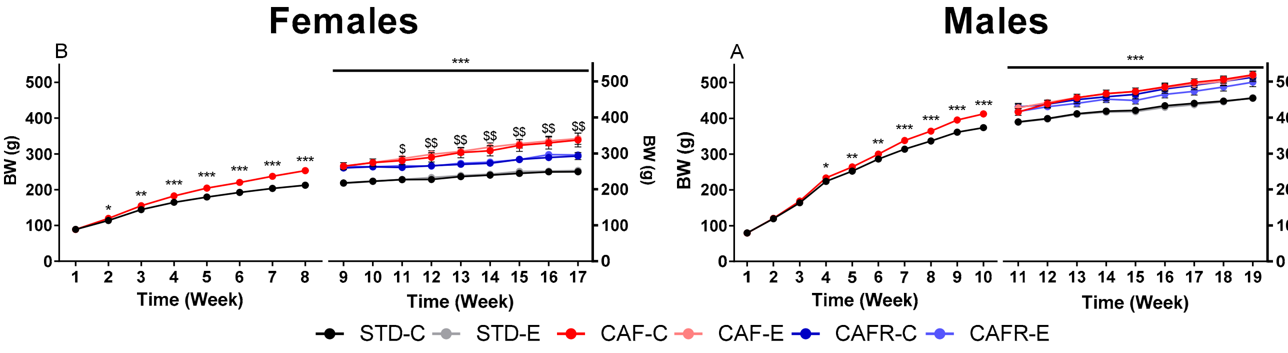

Supplementary Figure S1. Body weight progression in experiment 1 and 2. A. Body weight progression in females from the start of the experiment to the behavioural testing. B. Body weight progression in males from the start of the experiment to the behavioural testing. *p<0.05; **p<0.01; ***p<0.001 STD vs CAF; $p<0.05; $$p<0.01 CAF vs CAFR

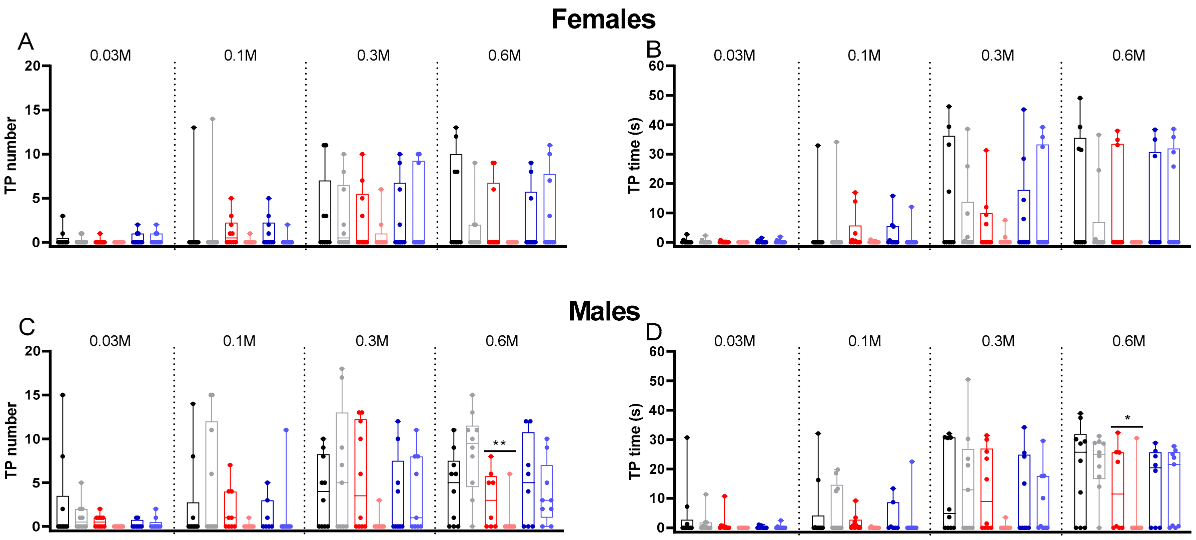

Supplementary Figure S2. Detail of the tongue protrusions (TP) number and time at each concentration. A. number of TP episodes in females at all concentrations tested. B. Time spent doing TP In females at all concentrations tested. C. number of TP episodes in males at all concentrations tested. D. Time spent doing TP In males at all concentrations tested. CAF feeding decreased the number of TP and the time spent doing TP in males at the 0.6M concentration [number TP: U=69, p=0.003; time TP: U=88, p=0.021]. * p<0.05; **p<0.01.

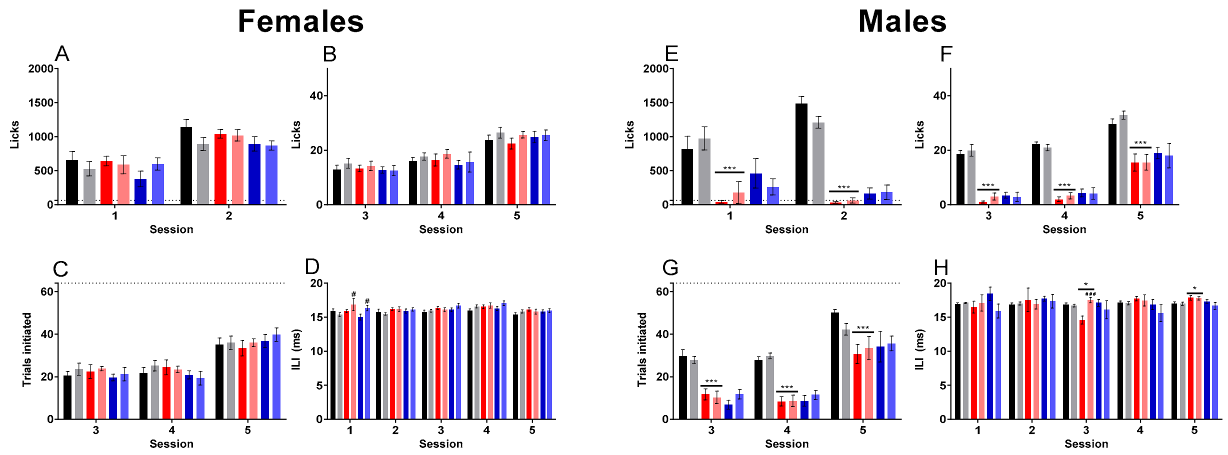

Supplementary Figure S3: Licking variables during the training in the brief-access licking test. A. Number of licks in S1 and S2 in females. B. Number of licks in S3, S4 and S5 in females. C. Number of trials initiated in S3, S4 and S5 in females. D. ILI times in S1-S5 in females. E. Number of licks in S1 and S2 in males. F. Number of licks in S3, S4 and S5 in males. G. Number of trials initiated in S3, S4 and S5 in males. H. ILI times in S1-S5 in males. Dashed lines in C and G indicate the total number of trials in a session (64 trials).

Statistical analysis of the parameters represented in Supplementary Figure 5 is detailed below:

In the training sessions in females the 1st analysis revealed no differences between the STD and CAF groups in total licks, trials initiated or licking latencies.

In the 2nd analysis we detected in session 1 that exercised animals showed longer ILI times than control animals ($p=0.027$). No effect of diet or the interaction was detected. No significant effect was detected for the total licks in session 1. No significant effects were detected for diet, exercise nor the interaction diet*exercise in total licks, trials initiated or licking latencies in sessions 2, 3, 4 or 5.

During the training sessions in males, the 1st analysis showed that STD consistently performed more licks to the spout than CAF (Session 1, S1: $p<0.001$; Session 2, S2: $p<0.001$; Session 3, S3: $p<0.001$; Session 4, S4: $p<0.001$ and Session 5, S5: $p<0.001$). No effect of exercise or the interaction diet*exercise were detected. STD also increased the number of trials initiated in sessions 3, 4 and 5 (S3: $p<0.001$; S4: $p<0.001$ and S5: $p<0.001$). No effect of exercise or the interaction diet*exercise were detected. No differences were detected in ILI times in sessions 1 and 2. In session 3 an effect of diet was detected in ILI time ($p<0.05$), with CAF animals performing shorter latencies, and of exercise ($p<0.001$), with exercised animals performing longer latencies, the interaction diet*exercise was significant ($p<0.001$), with CAF-E animals performing longer latencies than CAF-C animals, STD animals performed similar latencies regardless of exercise. In session 4 we detected a tendency for the factor diet ($p=0.096$), with CAF animals performing shorter latencies. The interaction diet*exercise was also close to significance ($p=0.079$), with CAF-E animals performing longer latencies than CAF-C animals, STD animals performed similar latencies regardless of exercise. No effects of exercise alone were detected in session 4. In session 5 we detected an effect of diet in ILI time ($p<0.05$), with CAF animals performing longer latencies than STD animals. No effect of exercise or the interaction diet*exercise was detected in session 5.

In the 2nd analysis comparing CAF and CAFR no differences were observed between groups in total licks, trials initiated or licking latencies.

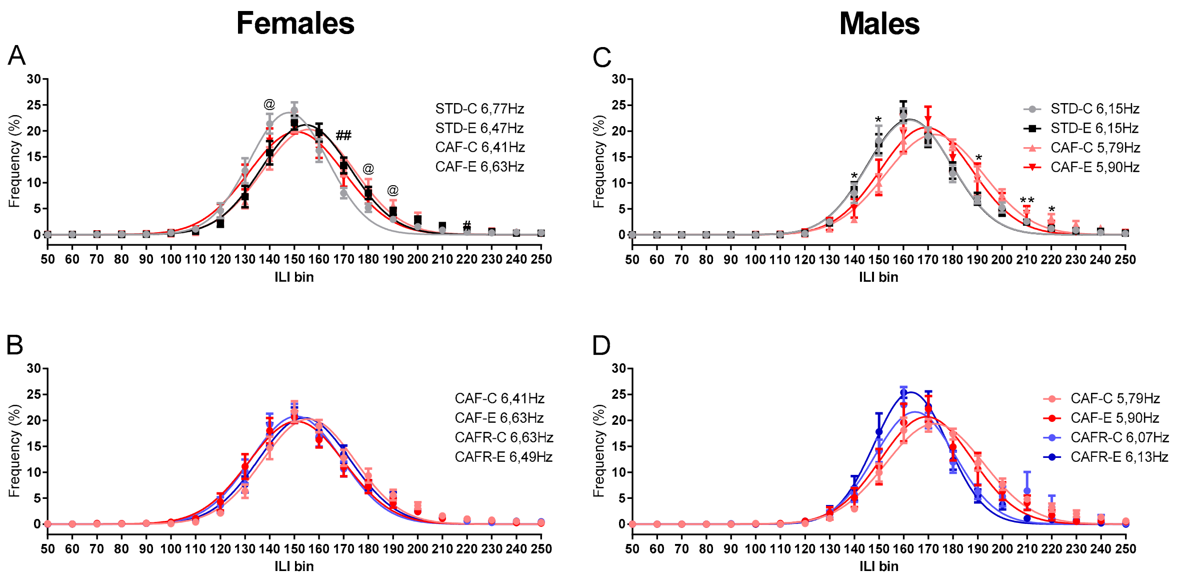

Supplementary Figure S4. Distribution of the interlick intervals (ILI) for all latencies between 50 and 250ms in duration. A. Distribution for STD and CAF females. B. Distribution for CAF and CAFR females. C. Distribution for STD and CAF males. D. Distribution between CAF and CAFR males. *p<0.05; **p<0.01 STD vs CAF; ##p<0.01 vs non-exercised corresponding diet group; @p<0.075 vs non-exercised corresponding diet group.

Statistical analysis of the parameters represented in Supplementary Figure 6 is detailed below:

We analysed the mean ILI distribution in the test session considering all ILI values between 50ms and 250ms. In the females the comparison between STD and CAF feeding revealed a main effect on ILI distribution ($p<0.001$). The diet and exercise factors were not significant by themselves, but the triple interaction ILI*diet*exercise showed a significance ($p<0.05$). Decomposition of this interaction showed that STD-E animals performed more ILIs in the 170 and 220ms bins ($p<0.05$), with 180, 190 and 210ms being close to significance [180ms: $p=0.058$; 190ms: $p=0.063$; and 210ms: $p=0.083$], while STD-C only tended to perform more ILIs in the 140ms bin ($p=0.072$). This was mirrored in the parameters of the Gaussian regression in which the mean of the regression was higher in STD-E than in STD-C (STD-C: 147ms±0.5; and STD-E: 154ms±0.6), suggesting that exercise in STD animals might shift the mean ILI distribution towards longer intervals between consecutive licks. CAF animals showed the same mean ILI distribution regardless of exercise. When comparing CAF and CAFR (Fig. 8.D) no effects were seen while the main effect of ILI distribution remained significant ($p<0.001$). Curve fit for the Gaussian regression was excellent with an R2 of 0.978±0.006.

When comparing STD and CAF feeding in males we found a main effect of the ILI distribution ($p<0.001$), and a significant ILI*diet interaction ($p<0.05$) indicating that CAF feeding changed the ILI distribution compared to STD. Pair wise comparisons between diets showed that STD-fed animals performed more ILIs in the 140 and 150 ms bins ($p<0.05$), while CAF-fed performed more ILIs in the 190, 210 and 220 ms bins ($p<0.05$), with 200 ms being close to significance ($p=0.081$). A similar result was found for the parameters of the Gaussian regression in which the mean of the regression was higher in CAF than in STD (CAF-C: 172ms±0.9; CAF-E: 169ms±0.7; STD-C: 162ms±0.7; and STD-E: 162ms±0.7), indicating that CAF feeding shifted the mean ILI distribution towards longer intervals between consecutive licks. Neither the exercise nor the interaction ILI*diet*exercise were significant. We then performed the 2nd analysis for the CAF and CAFR groups, which revealed no differences due to diet or exercise while the main effect of ILI distribution was maintained ($p<0.001$), indicating that CAFR animals maintained the same mean ILI distribution as CAF animals. Curve fit for the Gaussian regression was excellent with an R2 of 0.969±0.021.

Supplementary Table S1. Cafeteria (CAF) diet composition for the first period for experiment 1.

CAF Diet	Week 1	Week 4	Week 8
Ingredient (g/rat)			
Muffin	3	7	7
Bacon	3	6	7
Carrot	2.5	6	7
Biscuits with pâté	3.5	5	5
Biscuits with cheese	3.5	5	5
Jellied sugared milk	25	35	45
Chow	15	15	25
Total of diet provided (g/rat)	55.5	79	101
Total energy provided (Kcal/rat)	130	191	239

Supplementary Table S2. Detailed treadmill training protocol for the progressive intensity increase from session 1 until session 9 for experiment 1.

Time (min)	Intensity (m/min)						
	**S.1 - S.2**	**S.3**	**S.4**	**S.5**	**S.6**	**S.7 - S.8**	**S.9 onwards**
**0**	0	5	5	6	7	7	7
**1**	0	5	5	7	8	8	8
**2**	0	5	5	8	9	9	10
**3**	0	6	7	9	10	10	11
**4**	0	6	7	10	11	11	13
**5**	0	8	9	11	12	13	14
**6**	0	8	9	11	12	13	17
**7**	5	9	10	11	12	13	17
**8**	5	9	10	11	12	13	17
**9**	5	9	10	11	12	13	17
**10**	6	10	12	11	14	13	17
**11**	6	10	12	11	14	13	17
**12**	7	11	12	11	14	13	17
**13**	7	11	12	11	14	13	17
**14**	7	11	12	11	14	13	17
**15**	8	12	12	11	14	13	17
**16**	8	12	12	11	14	13	17
**17**	9	12	12	11	14	13	17
**18**	9	12	12	14	14	13	17
**19**	9	12	12	14	14	13	17
**20**	10	12	12	14	14	13	17
**21**	10	12	12	14	14	13	17
**22**	10	12	12	14	14	13	17
**23**	10	12	12	14	14	13	17
**24**	10	12	12	14	14	13	17
**25**	12	12	12	14	14	17	17
**26**	12	12	12	14	14	17	17
**27**	12	12	12	14	14	17	17
**28**	12	12	12	14	14	17	17
**29**	12	12	12	14	14	17	17
**30**	8	8	8	8	8	8	8
**31**	8	8	8	8	8	8	8
**32**	6	8	8	8	8	8	8
**33**	6	8	8	8	8	8	8
**34**	6	8	8	8	8	8	8
**35**	6	8	8	8	8	8	8
**Total distance (meters)**	241 m	351 m	365 m	411 m	433 m	457 m	519 m
